# Self-assembled nanostructured resistive switching memory devices fabricated by templated bottom-up growth

**DOI:** 10.1038/srep18967

**Published:** 2016-01-07

**Authors:** Ji-Min Song, Jang-Sik Lee

**Affiliations:** 1 Department of Materials Science and Engineering, Pohang University of Science and Technology (POSTECH), Pohang 790-784, Republic of Korea

## Abstract

Metal-oxide-based resistive switching memory device has been studied intensively due to its potential to satisfy the requirements of next-generation memory devices. Active research has been done on the materials and device structures of resistive switching memory devices that meet the requirements of high density, fast switching speed, and reliable data storage. In this study, resistive switching memory devices were fabricated with nano-template-assisted bottom up growth. The electrochemical deposition was adopted to achieve the bottom-up growth of nickel nanodot electrodes. Nickel oxide layer was formed by oxygen plasma treatment of nickel nanodots at low temperature. The structures of fabricated nanoscale memory devices were analyzed with scanning electron microscope and atomic force microscope (AFM). The electrical characteristics of the devices were directly measured using conductive AFM. This work demonstrates the fabrication of resistive switching memory devices using self-assembled nanoscale masks and nanomateirals growth from bottom-up electrochemical deposition.

Next-generation non-volatile memory needs fast operating speed, low power consumption, and good electrical reliability. Scaling is also very important for high-density data storage. Current candidates for next-generation non-volatile memory devices include phase change memory^1^, ferroelectric memory^2^, spin transfer torque-magnetic memory^3^, and resistive switching random access memory (ReRAM)^4^. Among these, ReRAM and the related memristors are promising candidates for next-generation non-volatile memory because of its simple metal–insulator–metal (MIM) structure and superior scalability. In addition, ReRAM has multilevel data storage capability. The memory density can be increased efficiently with multilevel data storage, resulting in reduction of production cost of ReRAM^5^,^6^,^7^,^8^.

In order to achieve high-density of ReRAM, the device needs to be fabricated at the nanoscale. Generally, lithographic techniques have been used to fabricate ordered nanostructures^9^,^10^,^11^, but these methods require a high production cost and a long processing time. Some non-conventional techniques have been introduced to overcome the problems. Specifically, nanoporous templates have been used because nanoscale materials/devices can be synthesized through physical vapor deposition of materials with nanoporous templates as the mask layer^12^. Among the various nanoporous templates, anodized aluminum oxide (AAO) has been developed as it has attractive features due to its good thermal and mechanical stability together with easy fabrication of wide range of pore diameters, inter-pore distances, and lengths of templates. Especially for the high aspect-ratio feature of the nanopores, AAO has been one of the best candidates that would appear as a useful template in industry as well as in academic research^13^,^14^.

Resistive switching behavior has been reported in various materials systems, including chalocogenides, perovskite oxides, and transition metal oxides (TMO). Recently, researchers have extensively studied TMO materials, such as NiO, TiO_2_, Al_2_O_3_, Nb_2_O_5_, as candidate materials for ReRAM, on account of the easy control of their chemical composition, low production cost, and compatibility with the conventional semiconductor processes^15^.

Typically, ReRAM devices with an MIM structure are fabricated by the lithography technique based on physical vapor deposition (PVD) methods. However, PVD methods are limited at the nanoscale due to clogging of the patterning masks by the deposited materials. Bottom-up approaches based on the self-assembled nano-templates have the potential to overcome these limits. The electrochemical deposition method is a representative bottom-up growth method. In this study, Ni bottom and top electrodes were deposited by the electrochemical deposition method. The electrodeposition of Ni from sulfamate electrolytes is an industrially important process. The sulfamate solutions are often preferred due to low deposited film stress, low sulfur content in the deposited metals, and the generally good mechanical properties of the Ni. Additionally, the high current efficiency of the bath typically allows for high deposition rates, permitting the rapid deposition of thick, low-stressed films^16^,^17^. The NiO layer formed by plasma oxidation on Ni substrate is employed to fabricate Ni/NiO/Ni ReRAM device, which shows a unipolar resistive switching property. Nanoscale non-volatile memory devices are demonstrated with simple fabrication processes for high-density device applications by bottom-up growth process at a low temperature.

## Results

**Figure 1:**
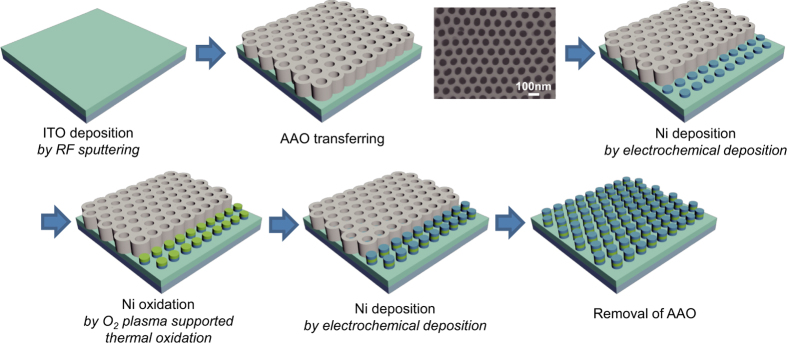
Schematic procedure for nano-scale ReRAM device fabrication. Planar SEM image of transferred AAO template is shown in the figure. The Ni is synthesized by electrochemical deposition with AAO as the template mask and finally array of MIM (Ni/NiO/Ni)-structured nanoscale ReRAM devices is fabricated.

Ordered nanoporous AAO templates synthesized by two-step anodization method are widely used to synthesize nanostructures such as nanorods and nanotubes^18^,^19^,^20^,^21^. In this study, AAO nanotemplates were used as the patterning mask for nanodot arrays, and the electrodes were fabricated by the electrochemical deposition method. During the first anodization of AAO, pores were generated randomly, so a uniform pore array was not obtained. The pores then began to grow in a direction perpendicular to the surface because of pore volume expansion associated with oxide growth. Thus, the uniformity of the AAO pore array depends on the elapsed time of the first anodization step. In this study, the first anodization was performed for 24 h; the layer formed in the first anodization step was then removed chemically. After the second anodization step, we obtained the uniform hexagonal shape of the AAO nanotemplate. In order to use the AAO template as the mask for nano-patterning, the AAO was filled with polystyrene (PS) and detached from the Al sheet. The barrier layer was chemically etched with phosphoric acid solution. The PS layer prevented the AAO templates from breaking during the transferring process and an increase in the pore size during the barrier layer removal. The PS/AAO layer was transferred onto the substrate. The PS layer was then removed by immersion in the propylene glycol methyl ether acetate (PGMEA) solution. The AAO template was confirmed by observation with a field emission scanning electron microscope (FE-SEM; JSM 7401F, JEOL). Figure 1 shows the schematic procedure to fabricate the ReRAM using bottom up processes.

**Figure 2:**
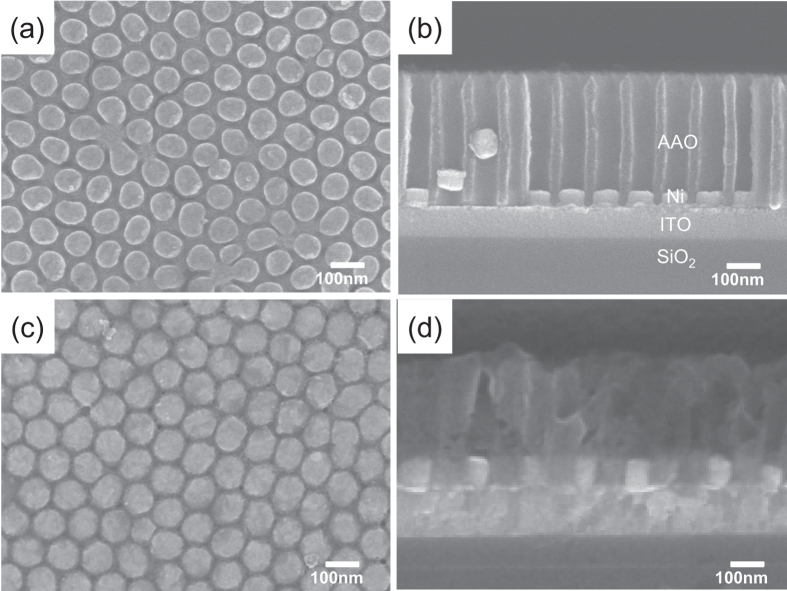
(**a**) Planar and (**b**) cross-sectional SEM images of electrochemically deposited Ni nanodots. (**c**) Planar and (**d**) cross-sectional SEM images of Ni/NiO/Ni structured nanodot ReRAM devices.

Figure 1 shows a planar view of the AAO template. The pore array was very well aligned with the hexagonal structure; the pore size was about 75 nm, the distance from pore to pore was about 100 nm, and the pore density was around 1 × 10^10 ^cm^−2^. As shown in Fig. 2 the AAO template had a depth of about 300 nm, and it grew in the direction perpendicular to the substrate. The depth of the AAO template can be easily controlled by controlling the second anodization time. With this technique, patterning at a scale of less than 100 nm can be easily achieved, and it can be applied to other processes that require nanosized structures. The pore size and density of AAO nanotemplates can be controlled by changing the electrolyte solution and anodization voltage^22^,^23^. Thus, it is a versatile method for making nanopatterns without using optical lithography techniques.

Electrochemical deposition is a conventional deposition method. By using this method metal deposition can be conveniently achieved with a simple solution process, but without a vacuum deposition process. In this study, electrochemical deposition was adopted to deposit Ni nanodots. Ni electrochemical deposition is similar to other electrochemical deposition processes; that is, direct current is applied to make flow between two electrodes immersed in aqueous solution of Ni salts. The flow of direct current causes the cathode to become covered with metallic Ni. The nickel is in the form of divalent, positively charged ions (Ni^2+^) in solution. The positive Ni^2+^ ions react with 2e^−^ and are converted to metallic nickel (Ni^0^) at the cathode surface. The nickel ions discharged at the cathode are thus replenished by those formed at the anode^24^. Electrochemical deposition was performed under the previously given conditions using the transferred AAO nanotemplate. As shown in Fig. 2(a,b), the Ni nanodots formed uniformly, and the pattern was well aligned with the hexagonal-packed structure as in the AAO templates. The thickness of the Ni layers that formed on the ITO layer by electrochemical deposition for 1 min was 40 nm (Fig. 2(b)). Another advantage of electrochemical deposition is that the materials grow through the AAO from the bottom of the template. Because of this advantage, the nanosized mask is not obstructed with materials during deposition. In contrast, PVD methods such as sputtering are limited because nanosized masks become clogged with deposited materials. Thus, there is a limit in fabricating nanoscale devices by the PVD method.

After forming the Ni layer, O_2_ plasma-supported thermal oxidation was performed to make the NiO resistive switching layer at low temperature. There are several techniques to prepare NiO layer, such as thermal oxidation^25^,^26^, anodic oxidation^27^,^28^, thermal spray^29^, and plasma oxidation. O_2_ plasma-supported oxidation has several advantages over other oxidation techniques, e.g., fast oxidation capability, low temperature process, and ease of forming uniform and dense layer^30^,^31^. NiO layers are generally formed at high temperatures above 400 °C^32^, but such a high-temperature process cannot be applied to plastic substrate-based flexible devices. In order to overcome this limitation, we attempted O_2_ plasma-supported oxidation at 180 °C.

**Figure 3:**
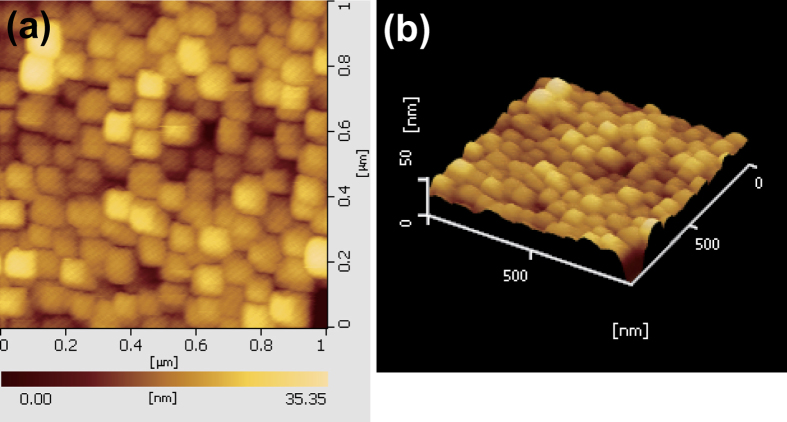
AFM images of Ni/NiO/Ni structured nanodots. (**a**) Topography of Ni/NiO/Ni nanodots and (**b**) 3-dimensional AFM image of Ni/NiO/Ni nanodots.

After NiO formation, electrochemical deposition was again carried out to form the Ni layer as the top electrode. Once, the NiO layer is formed on the Ni surface, resistance of the surface is increased. So, this step was performed at a higher voltage condition than the previous electrochemical deposition step to apply same current density. The completed devices were examined with an FE-SEM and contact-mode atomic force microscope (AFM; SPA 400 microscope, SEIKO). MIM structured nanodot devices were fabricated with a high density of 1 × 10^10 ^cm^−2^ (Fig. 2(c)). Nanodot array devices having approximately 80 nm height formed uniformly on the substrate (Fig. 2(d)). The average size of the nanodot devices was 5.02 × 10^−11 ^cm^2^; the device size can be controlled by controlling the pores sizes^12^. Figure 3 shows AFM images of the Ni/NiO/Ni structured nanodot array. The scan rate was 0.5 Hz, the scan configuration was 256 × 256 pixels, and the scan size was 1 × 1 μm^2^. It is confirmed that the nanodot array was formed uniformly. The complex nanoscale structures were successfully synthesized without any lithography tools, and all procedures were performed at low temperatures.

**Figure 4:**
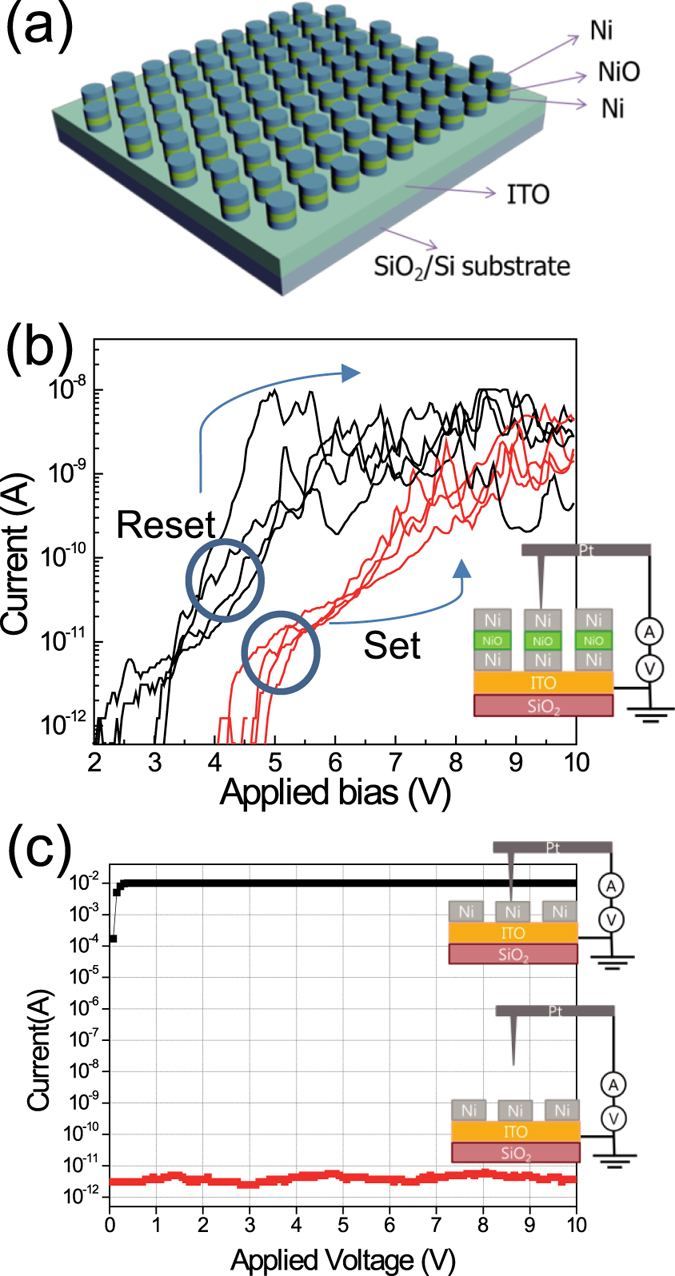
(**a**) Schematic illustration of fabricated nanoscale ReRAM device for electrical measurement. (**b**) The resistive switching characteristic of nanoscale ReRAM device. The nanoscale memory device can be repeatedly set and reset by applied electrical biases. The memory device showed unipolar resistive switching behaviour. (**c**) The current–voltage responses of the sample with an AFM probe that was short circuit state or open circuit state to determine the validity of using the AFM probe for electrical biasing.

To confirm that the nanopatterned ReRAM devices were working properly, the electrical characteristics of the ReRAM devices were measured with conductive atomic force microscopy (CAFM) (Fig. 4). The current–voltage responses were measured with an AFM probe that was directly attached to the bottom electrode (short circuit) and without contacting the top electrode (open circuit) to verify the proper act of AFM probe as the bias applying tip^12^. In the result, a current noise level of about 10^−12 ^A was measured at open circuit. In the case of short circuit, the current immediately increased up to the compliance level (10 mA). This verified that the CAFM probe can be used as an electrical conducting probe, and electrical properties that were measured with the CAFM probe occurred in the nanostructured ReRAM devices as well^12^.

In this study, the set voltage (V_Set_) was defined as the critical voltage at which the devices transformed from the high-resistance state (HRS) to the low-resistance state (LRS); the reset voltage (V_Reset_) was defined as the critical voltage for the opposite transformation. NiO is reported to show unipolar or bipolar switching behavior according to the electrodes, compositions, process conditions, etc^33^,^34^. In our case, unipolar resistive switching property was observed (Fig. 4(b)). The voltage was swept from 0 V to 10 V, and the current compliance was fixed at 10^−8 ^A to prevent the devices from breakdown. The set voltage (V_Set_) was nearly 9 V, and the reset voltage (V_Reset_) was about 6 V. The LRS and HRS could be clearly discerned. The highest current level of LRS was about 10^−8 ^A, and the LRS/HRS current ratio was about 10 to 100. These results verified that nanopatterned devices were successfully fabricated. On/off ratio of the resistance state is directly related to the sensing margin of memory devices. High on/off ratio is required for multilevel data storage and reliable device operation. Our device shows on/off ratio of ~100. It is reported that operation of high-density resistive switching memory device with on/off ratio of ~10 was successfully demonstrated by optimization of read scheme to reduce the sneak current^35^. Therefore, it is thought that the memory device fabricated by bottom-up processes can be used as the memory element in real device applications. Anyway, it is very important to get high on/off ratio for integration strategy, so the optimization of device structure and materials will be done for high on/off ratio. The set and reset phenomena occurred repeatedly. In this work the set/reset voltages seem to be high compared to other resistive switching memory devices. The reason to have high set/reset voltages is thought to be due to the thickness of NiO formed through the oxidation of bottom Ni layer. Optimization of oxidation process to form NiO will improve the quality of NiO, resulting in improvement of electrical properties. We measured the endurance property of resistive switching memory device with a structure of Ni/NiO/Ni (Supplementary Fig. S1(a)). Although there are some fluctuations in LRS and HRS current levels on/off ratio of higher than 10 is maintained. In our case, electrical properties such as V_Set_, V_Reset_, and resistances of LRS and HRS showed some variation, but the devices worked repeatedly. To confirm the device uniformity statistical cumulative probability of current levels (HRS and LRS) is determined (Supplementary Fig. S1(b)). Some non-uniform property is observed, but considering the fact that all devices were fabricated using only bottom-up growth without any vacuum deposition and lithographic tools we believe the process developed in this study has a good potential to be used in real memory device fabrication with further refining the process developed in this study.

The electrical properties of ReRAM are mostly related to the resistive switching materials and electrodes. Some studies have reported that the electrical properties are also related with the thickness of the switching materials^36^,^37^,^38^. The investigation of resistive switching behaviour with different thicknesses of the switching materials and/or with different electrode materials will be very important. Further study is under the way to investigate the switching characteristics according to changes in the switching materials and electrodes. In our devices unipolar switching is observed (Fig. 4(b)), so the filament formation and rupture are thought to be the main cause of resistive switching^34^,^39^. In case of unipolar switching the filament formation is determined by stochastic processes, so fluctuation in set/reset processes reportedly happens^39^. Schematic illustration to explain the switching mechanism is shown in Supplementary Fig. S2. In this study, we showed that nanoscale resistive-switching-memory devices could be fabricated by a facile bottom-up process without using conventional lithography method. In addition, low temperature oxidation was achieved using O_2_ plasma-supported oxidation.

Most electrical devices continue to require smaller sizes and more flexibility with a uniform array. Some researchers have examined the synthesis of well-ordered AAO templates by controlling the pore nucleation site. Experiments that apply AAO and flexible substrates are already ongoing. In the near future, well-ordered and flexible nanoscale electrical devices will be realized by the use of optimized AAO nanotemplates and low-temperature processes.

## Discussion

We made AAO nanotemplates by using the two-step anodization method. Ni/NiO/Ni structured ReRAM devices that were less than 100 nm in size were fabricated through the AAO nanotemplate. Ni nanodots were synthesized by the electrochemical deposition method with a high density and uniformity. The NiO layer was formed at low temperature through O_2_-plasma-enhanced thermal oxidation. The structures of fabricated memory devices were analyzed with FE-SEM and AFM. The results confirmed that nanoscale memory devices were fabricated only utilizing bottom-up processes. Fabricated ReRAM devices had a density of 1 × 10^10^/cm^2^; each cell was separated from each other. The electrical properties of the devices were measured by using CAFM. The results showed unipolar resistive switching characteristics, and the LRS and HRS were clearly distinct from each other. This nano-patterning technique can easily be applied to fabrication of functional nanoelectronic devices, and the low temperature oxidation method can be applied to flexible device fabrication. This study has a great potential to be applied to manufacturing nanoscale, high-density non-volatile memory devices in the future.

## Methods

### Fabrication of AAO masks

In this study, we adopted the two-step anodization method to produce anodic aluminum oxide (AAO) templates with uniform pores^40^. First, aluminum foil (99.999%, Goodfellow) was prepared to fabricate the AAO template. Prior to anodization, the Al foils were sequentially immersed in ethanol (C_2_H_5_OH), acetone (CH_3_COCH_3_), and deionized water (DI water) to remove impurities such as organics that are attached to the surface, and they were then ultrasonicated for 15 min each. In order to produce the AAO template, water around the reaction vessel was circulated using a chiller to maintain a constant temperature. The aluminum foil was electropolished at 18 V and 7 °C in the mixed solution of perchloric acid (HClO_4_) and ethanol (1:5 volume fraction) for 5 min to make a smooth surface and rinsed in DI water. We then performed the two-step anodization method. A solution of 0.3 M oxalic acid (C_2_H_2_O_4_) was used as the electrolyte solution for the first and second anodization. Electropolished Al foil was anodized at 40 V and 15 °C for 24 h. In the first anodization, AAO was chemically etched by a mixture of chromic acid (1.6 wt%, H_2_CrO_4_) and phosphoric acid (6 wt%, H_3_PO_4_) at 60 °C. The second anodization was performed under the same conditions of the first anodization for 4 min. The AAO template was then immersed in 0.1 M phosphoric acid at 30 °C to make the pores wider and more uniform. After the widening process, the AAO template was filled with polystyrene solution (1.6 wt% PS/CHCl_3_) and heated at 80 °C for 2 h to dry the solvent^41^. The AAO/PS layer was separated from the Al foil by immersion in mercury chloride (HgCl_2_) supersaturated solution for several hours and then rinsed with DI water. The AAO template separated from the Al foil was packed with a barrier layer being used as a mask; this had to be removed from the AAO. The AAO/PS was immersed in a solution of 0.1 M phosphoric acid at 30 °C for 30 min to remove the barrier layer. The AAO/PS was transferred onto a substrate and then annealed at 100 °C for 30 min. After adsorption, the PS layer was removed from the AAO by immersion in PGMEA^12^.

### Fabrication of Ni/NiO/Ni structured nanodot ReRAM

Figure 1 shows the fabrication of the Ni/NiO/Ni structured nanodot array. Indium tin oxide (ITO) deposited on SiO_2_ substrate was used as the working electrode for electrochemical deposition. In order to remove dirt on the surface, the SiO_2_ substrate was immersed in 100 °C of piranha solution (4:1 = H_2_O_2_:H_2_SO_4_ volume) for 10 min. It was then rinsed with DI water. After cleaning, a 100-nm-thick layer of ITO (In_2_O_3_:SnO_2_ = 9:1) was deposited onto the SiO_2_ substrate by radio frequency (RF) magnetron sputtering with 50 W power at room temperature; the base pressure was 3 × 10^−6 ^Torr, and the working pressure was 3 × 10^−3 ^Torr. The AAO template was transferred to a substrate. The Ni electrode was then deposited by the electrochemical deposition method using the transferred AAO template. A mixture of nickel chloride (NiCl_2_∙6H_2_O), nickel sulfamate (Ni(H_2_NSO_3_)_2_), boric acid (H_3_BO_3_), and sodium acetate (CH_3_COONa) was titrated to pH of 3.4 by sulfuric acid (H_2_SO_4_) at 10 °C; the mixture was used as the electrolyte solution for electrochemical deposition^20^. The ITO substrate and carbon rod were used as the working and counter electrodes, respectively. Ni deposition was performed under the conditions of 4 mA/cm^2^ and 10 °C for 1 min. The NiO layer was formed on the surface of each Ni nanodot by O_2_ plasma-supported thermal oxidation at 250 W of O_2_ plasma and 180 °C for 1 h. Electrochemical deposition of Ni was again performed in order to make the top electrode for the ReRAM device under the conditions of 4 mA/cm^2^ and 10 °C for 1 min. After deposition, the AAO mask was removed by the taping method.

## Additional Information

**How to cite this article**: Song, J.-M. and Lee, J.-S. Self-Assembled Nanostructured Resistive Switching Memory Devices Fabricated by Templated Bottom-up Growth. *Sci. Rep.* **6**, 18967; doi: 10.1038/srep18967 (2016).

## Supplementary Material

Supplementary Information

## References

[b1] Hamann H. F., O’Boyle M., Martin Y. C., Rooks M. & Wickramasinghe H. K. Ultra-high-density phase-change storage and memory. Nature Mater. 5, 383–387 (2006). 16604077 10.1038/nmat1627

[b2] Rana D. S. *et al.* Understanding the Nature of Ultrafast Polarization Dynamics of Ferroelectric Memory in the Multiferroic BiFeO_3_. Adv. Mater. 21, 2881–2885 (2009).

[b3] Halupka D. *et al.* In Solid-State Circuits Conference Digest of Technical Papers (ISSCC), 2010 IEEE International 256–257 (2010).

[b4] Russo U., Ielmini D., Cagli C. & Lacaita A. L. Filament Conduction and Reset Mechanism in NiO-Based Resistive-Switching Memory (RRAM) Devices. IEEE Trans. Electron Devices 56, 186–192 (2009).

[b5] Yoshida C., Tsunoda K., Noshiro H. & Sugiyama Y. High speed resistive switching in Pt∕TiO_2_∕TiN film for nonvolatile memory application. Appl. Phys. Lett. 91, 223510 (2007).

[b6] Terai M., Sakotsubo Y., Kotsuji S. & Hada H. Resistance Controllability of Ta_2_O_5_/TiO_2_ Stack ReRAM for Low-Voltage and Multilevel Operation. IEEE Electron Device Lett. 31, 204–206 (2010).

[b7] Kannan V., Senthilkumar V. & Rhee J. K. Multi-level conduction in NiO resistive memory device prepared by solution route. J. Phys. D: Applied Physics 46, 095301 (2013).

[b8] Ielmini D., Cagli C., Nardi F. & Zhang Y. Nanowire-based resistive switching memories: devices, operation and scaling. J. Phys. D: Applied Physics 46, 074006 (2013).

[b9] Ballav N., Schilp S. & Zharnikov M. Electron-Beam Chemical Lithography with Aliphatic Self-Assembled Monolayers. Angew. Chem. Int. Ed. 120, 1443–1446 (2008). 10.1002/anie.200704105 18188856

[b10] Stamou D. *et al.* Site-Directed Molecular Assembly on Templates Structured with Electron-Beam Lithography. Langmuir 20, 3495–3497 (2004). 15875370 10.1021/la049954j

[b11] Bhuvana T. & Kulkarni G. U. Highly Conducting Patterned Pd Nanowires by Direct-Write Electron Beam Lithography. ACS Nano 2, 457–462 (2008). 19206570 10.1021/nn700372h

[b12] Lyu S.-H. & Lee J.-S. Highly scalable resistive switching memory cells using pore-size-controlled nanoporous alumina templates. J. Mater. Chem. 22, 1852 (2012).

[b13] Jung J. S. *et al.* Electrodeposited Nickel Nanodots Array on the Silicon Wafer. Bulletin of the Korean Chemical Society 29, 2169–2171 (2008).

[b14] Park M.-S., Yu G.-D. & Shin K.-S. Alumina Templates on Silicon Wafers with Hexagonally or Tetragonally Ordered Nanopore Arrays via Soft Lithography. Bulletin of the Korean Chemical Society 33, 83–89 (2012).

[b15] Lee J.-S. Progress in non-volatile memory devices based on nanostructured materials and nanofabrication. J. Mater. Chem. 21, 14097–14112 (2011).

[b16] Kelly J. J., Goods S. H., Talin A. A. & Hachman J. T. Electrodeposition of Ni from Low-Temperature Sulfamate Electrolytes. J. Electrochem. Soc. 153, C318 (2006).

[b17] Feng H.-P. *et al.* Nanoparticle-Enabled Selective Electrodeposition. Adv. Mater. 23, 2454–2459 (2011). 21538987 10.1002/adma.201004656

[b18] Zhi L., Wu J., Li J., Kolb U. & Mullen K. Carbonization of disclike molecules in porous alumina membranes: toward carbon nanotubes with controlled graphene-layer orientation. Angew. Chem. Int. Ed. 44, 2120–2123 (2005). 10.1002/anie.200460986 15736234

[b19] Hurst S. J., Payne E. K., Qin L. & Mirkin C. A. Multisegmented One-Dimensional Nanorods Prepared by Hard-Template Synthetic Methods. Angew. Chem. Int. Ed. 45, 2672–2692 (2006). 10.1002/anie.200504025 16570332

[b20] Lee W., Scholz R., Nielsch K. & Gösele U. A Template-Based Electrochemical Method for the Synthesis of Multisegmented Metallic Nanotubes. Angew. Chem. Int. Ed. 44, 6050–6054 (2005). 10.1002/anie.200501341 16124018

[b21] Martinson A. B. F., Elam J. W., Hupp J. T. & Pellin M. J. ZnO nanotube based dye-sensitized solar cells. Nano Lett. 7, 2183–2187 (2007). 17602535 10.1021/nl070160+

[b22] Masuda H., Yada K. & Osaka A. Self-ordering of cell configuration of anodic porous alumina with large-size pores in phosphoric acid solution. Jpn. J. Appl. Phys. Part 2 - Lett. 37, L1340–L1342 (1998).

[b23] Shingubara S., Morimoto K., Sakaue H. & Takahagi T. Self-Organization of a Porous Alumina Nanohole Array Using a Sulfuric/Oxalic Acid Mixture as Electrolyte. Electrochem. Solid-State Lett. 7, E15 (2004).

[b24] Brankovic S. R., Vasiljevic N. & Dimitrov N. Modern Electroplating V (eds Paunovic M. & Schlesinger M.) 573–616 (Wiley, New York, 2010).

[b25] Jeurgens L. P. H., Sloof W. G., Tichelaar F. D. & Mittemeijer E. J. Composition and chemical state of the ions of aluminium-oxide films formed by thermal oxidation of aluminium. Surf. Sci. 506, 313–332 (2002).

[b26] Jeurgens L. P. H., Sloof W. G., Tichelaar F. D. & Mittemeijer E. J. Structure and morphology of aluminium-oxide films formed by thermal oxidation of aluminium. Thin Solid Films 418, 89–101 (2002).

[b27] Vrublevsky I., Parkoun V., Schreckenbach J. & Marx G. Effect of the current density on the volume expansion of the deposited thin films of aluminum during porous oxide formation. Appl. Surf. Sci. 220, 51–59 (2003).

[b28] Asoh H., Matsuo M., Yoshihama M. & Ono S. Transfer of nanoporous pattern of anodic porous alumina into Si substrate. Appl. Phys. Lett. 83, 4408 (2003).

[b29] Vippola M., Vuorinen J., Vuoristo P., Lepisto T. & Mantyla T. Thermal analysis of plasma sprayed oxide coatings sealed with aluminium phosphate. J. European Ceram. Soc. 22, 1937–1946 (2002).

[b30] Mozetic M., Zalar A., Cvelbar U. & Babic D. AES characterization of thin oxide films growing on Al foil during oxygen plasma treatment. Surface and Interface Analysis 36, 986–988 (2004).

[b31] Hoey M. L., Carlson J. B., Osgood R. M., Kimball B. & Buchwald W. RF plasma oxidation of Ni thin films sputter deposited to generate thin nickel oxide layers. Appl. Phys. Lett. 97, 153104 (2010).

[b32] López-Beltrán A. M. & Mendoza-Galván A. The oxidation kinetics of nickel thin films studied by spectroscopic ellipsometry. Thin Solid Films 503, 40–44 (2006).

[b33] Akinaga H. & Shima H. Resistive Random Access Memory (ReRAM) Based on Metal Oxides. Proc. IEEE 98, 2237–2251 (2010).

[b34] Sawa A. Resistive switching in transition metal oxides. Mater. Today 11, 28–36 (2008).

[b35] Lee H. D. *et al.* Integration of 4F2 selector-less crossbar array 2Mb ReRAM based on transition metal oxides for high density memory applications. in 2012 Symposium on VLSI Technology (VLSIT) 151-152 (10.1109/VLSIT.2012.6242506) (12–14 June 2012).

[b36] Terai M., Sakotsubo Y., Saito Y., Kotsuji S. & Hada H. Effect of bottom electrode of ReRAM with Ta_2_O_5_/TiO_2_ stack on RTN and retention. In 2009 IEEE International Electron Devices Meeting (IEDM) 1–4 (10.1109/IEDM.2009.5424226) (7–9 Dec. 2009).

[b37] Kim W.-G. & Rhee S.-W. Effect of the top electrode material on the resistive switching of TiO_2_ thin film. Microelectronic Engineering 87, 98–103 (2010).

[b38] Oh S. C., Jung H. Y. & Lee H. Effect of the top electrode materials on the resistive switching characteristics of TiO2 thin film. J. Appl. Phys. 109, 124511 (2011).

[b39] Goux L. *et al.* Coexistence of the bipolar and unipolar resistive-switching modes in NiO cells made by thermal oxidation of Ni layers. J. Appl. Phys. 107, 024512 (2010).

[b40] Masuda H. & Fukuda K. Ordered metal nanohole arrays made by a 2-step replication of honeycomb structures of anodic alumina. Science 268, 1466–1468 (1995). 17843666 10.1126/science.268.5216.1466

[b41] Lee W. *et al.* Individually addressable epitaxial ferroelectric nanocapacitor arrays with near Tb inch^−2^ density. Nature Nano. 3, 402–407 (2008). 10.1038/nnano.2008.161 18654563

