# Supplementary material for: Self-assembled nanostructured resistive switching memory devices fabricated by templated bottom-up growth
Source: Sci Rep. 2016 Jan 7;6:18967. doi: 10.1038/srep18967 (PMC4704057; doi:10.1038/srep18967)
Supplement: Supplementary Information [file srep18967-s1.pdf]

Supplementary Information

Self-assembled nanostructured resistive switching memory devices fabricated by templated bottom-up growth

Ji-Min Song & Jang-Sik Lee

Department of Materials Science and Engineering, Pohang University of Science and Technology (POSTECH), Pohang 790-784, Republic of Korea.

Correspondence and requests for materials should be addressed to J.S.L. (email: jangsik@postech.ac.kr)

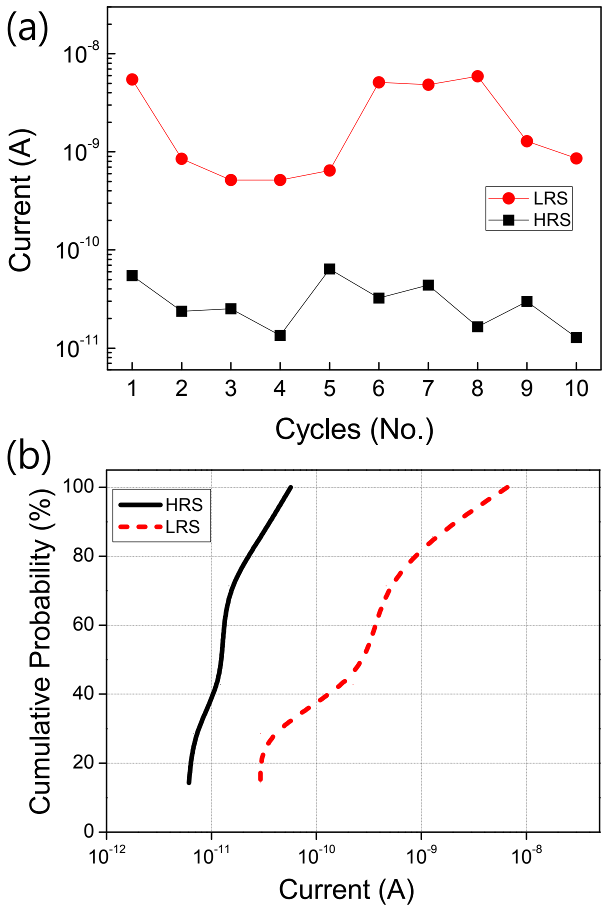

Supplementary Figure S1. (a) Endurance property of resistive switching memory device with a structure of Ni/NiO/Ni. (b) Statistical cumulative distribution of HRS and LRS currents.

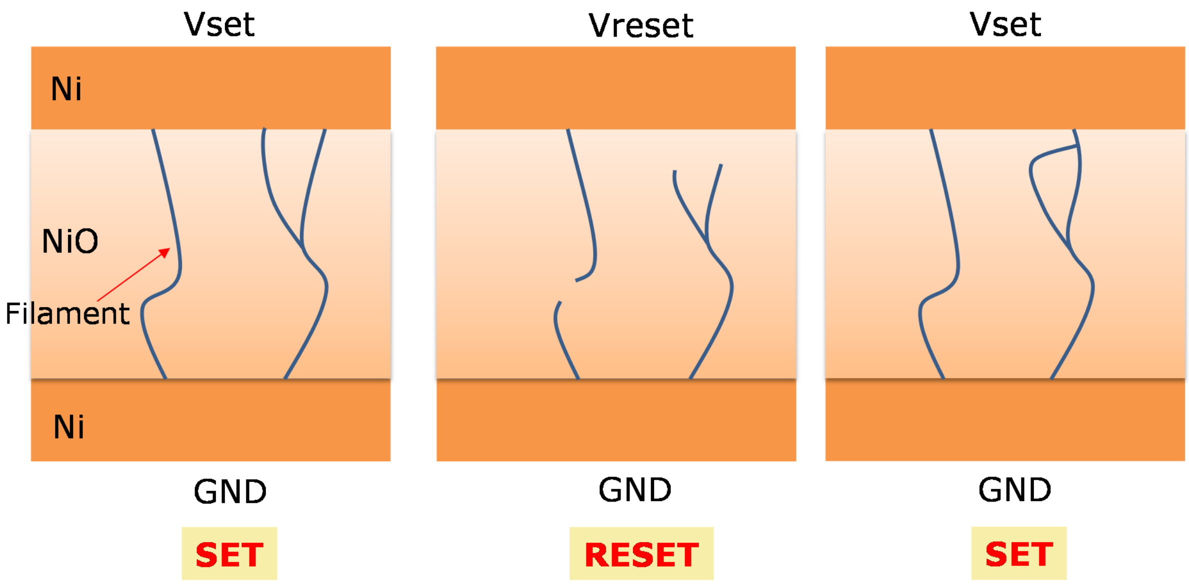

Supplementary Figure S2. Schematic illustration of set and reset processes in Ni/NiO/Ni structure showing unipolar resistive switching. The concept is adopted from Goux et al., J. Appl. Phys. 107, 024512 (2010).
